# Phase 0 Workshop at the 20th EORTC-NCI-AACR Symposium, Geneva

**DOI:** 10.3332/ecancer.2008.107

**Published:** 2008-11-12

**Authors:** S Camporesi

**Affiliations:** European School of Molecular Medicine, IFOM-IEO Campus, Via Adamello, 16, 20139 Milan, Italy

## Are phase 0 clinical trials really necessary?

This question was posed by James Doroshow (National Cancer Institute, Bethesda, USA) at the 20th EORT-NCI-AARC Symposium on Molecular Targets and Cancer Therapeutics (Geneva, 21–24 October 2008). Together with E Leo (Beerse, Belgium), James Doroshow—since 2004 Director of the Division of Cancer Treatment and Diagnosis at NCI—was co-chairing the workshop on phase 0 clinical trials. In his introduction to the speakers, Doroshow raised six open questions, namely whether phase 0 trials are useful, ethically acceptable, feasible, whether they speed up the drug development process and save money, and whether there is room for improvement.

In the Critical Path Report published in March 2004, the US Food and Drug Administration denounced the ‘slowdown, instead of expected acceleration, in innovative medical therapies reaching patients’ [1]. Following this concept, in January 2006, the FDA published new industry guidelines for early exploratory drug studies (i.e. phase 0 studies) in humans [2].

In 2003, the European Agency for the Evaluation of Medicinal Products (EMEA) had issued a similar concept note, followed by a Position Paper on the non-clinical safety studies needed to support human clinical trials, using microdosing techniques [3]. As defined by EMEA and FDA guidelines, phase 0 trials are early first-in-human clinical studies, conducted before the traditional dose escalation, safety and tolerance phase 1 studies.

According to both regulatory agencies, phase 0 trials are necessary, since current strategies for drug development are still predominantly based on the assumption that the investigational agent has a dose–toxicity relationship, and that efficacy is somewhat related to toxicity, but such assumptions may not be valid for new molecularly targeted agents [4]. For such new drugs, there is need to alter the traditional drug-development sequence, where exploratory phase 0 trials should be designed that focus on extensive agent characterization and target-assay development, including molecular imaging studies, in a limited number of patients (10–15), who will each be exposed to a limited number of doses of the study agent (less than two weeks) [2,3].

Phase 0 trials have no therapeutic intent and, at least in principle, should eliminate therapeutic failures early in the cancer drug development for agents without biological effectiveness, thus reducing costs and time of drug development [4,5].

According to C Garner (Xceleron Ltd, Heslington York, UK) humans are ‘the best model for humans’, and the excessive weight put on animal models for cancer drug development must be shifted in the direction of phase 0 trials. ‘The poor correlation between animal and human bioavailability’, Garner said, can be represented in a chart that ‘looks like stars in the sky’. Instead of using animals for cancer drug modelling, ‘you may as well toss a coin’, he also added provocatively [6]. Garner then dwelled on the technical details of AMS (accelerator mass spectrometry) as a tool for phase 0 microdose studies to understand human anatomy and drug bioavailability [7,8]. Microdose studies are one of the major applications of phase 0 trials, as illustrated by FDA and EMA guidelines [2,3], which define a microdose as less than 1/100th of the dose of the test substance calculated from *in vitro* and animal models to yield a pharmacological effect, with a maximum dose ≤ 100 μg [3]. AMS separates C isotopes on the basis of mass and energy differences and allows the study of the metabolic pathways of a 14C labelled drug and its metabolites.

Because of the AMS huge scale sensitivity, up to 10-18 -10-21 g, the radiation hazard for the patient becomes negligible. As a consequence, clinical studies with AMS do not require extensive pre-clinical animal toxicology work. This trend is in line with the FDA and EMEA guidelines, which for microdose studies require only extended single-dose toxicity studies in both genders of a single mammalian species to establish a dose inducing a minimal toxic effect or a safety margin [2,3]. In conclusion, Garner remarked how phase 0 microdose studies are definitely useful for providing early information about human absorption, distribution, metabolism and excretion (ADME) data; for comparing ADME parameters for several drug candidates and choosing the lead molecule to take on and develop, and for assisting in selecting the first dose for a subsequent phase 1 study. In his experience, phase 0 studies have a short, six-month timeframe from the bench to the bedside and do speed up the drug development process while helping to determine the approximate cost of the drug [6]. One obstacle may be represented by the pharmacokinetic profile of the drug, as a microdose may not predict the behaviour of clinical doses if there is no linear PK. Drugs with a linear or near linear PK—which constitute about 70% of the totality of the candidates according to Garner [6]—should be chosen as candidates for phase 0 studies.

‘Change we need’ was Jeremy Collins’ (National Cancer Institute, Rockville, MD, USA) opinion on the cancer drug development process and the US political situation. The phase 0 concept is ‘not simply a small incremental change in the cancer drug development process’, but is a drastic ‘re-engineering of the drug pipeline’ [9]. According to Collins, phase 0 cancer trials require a role reversal in goal priority, as cancer trials until the 1990s had as primary goal toxicity determination, and as secondary activity determination, while phase 0 studies have as primary goal pharmacodynamics assessment (as occupancy of the receptor by the compound, determination of the inhibition of the target), as secondary pharmacokinetics (as the extent of absorption of half life of the drug in the body), and toxicity is not expected [10]. In his talk, Collins focused on the applications of phase 0 trial for establishing in humans a ‘molecular proof of concept’, which include an evaluation of the biological effects on the target in tumour biopsies or other surrogates (such as peripheral blood mononuclear cells) and a determination of the PD and PK of the drug. In such applications, a pharmacological concentration of the drug can be (briefly) achieved. Collins also mentioned the importance of imaging studies to have a functional relevance and not be merely ‘decorations’ to a study, [11] as they should inform on the reception and/or compound biodistribution. To be useful, phase 0 trials must be able to answer fundamentally important ‘stop/go’ questions, such as: Does the drug hit the target?

The raising of such questions necessitates a redesign of the information flow in the drug development process in order to find a different proof of concept to facilitate decision making. As an example, Collins talked about the first NCI phase 0 trial with the PARP inhibitor (ABT-888) done in collaboration with Abbott industries, where two fundamentally stop/go questions were answered positively, thus permitting the continuation of the drug development: Can the target plasma concentration be achieved orally? And can tumour biopsies give definitive results with a single dose [12,13]? The requirement of multiple biopsies for patients in trials with no direct benefits is ethically problematic, as remarked by a discussion that followed Collins’ speech, and valid alternatives are actively searched for, such as imaging, peripheral blood mononuclear cells and circulating tumour cells. Phase 0 cancer trials are unique in having no therapeutic intent at all, and the ethical solution will probably not look like a wide comprehensive blanket covering all trials of this kind, but as a case-by-case justification.

G Gordon (Abbott Oncology Development, USA) gave his industry perspective to the experience of the first phase 0 trial conducted together with the NCI on the ABT-888 molecule [13], where information from the phase 0 study provided informational guidance for subsequent phase 1 combination trials with FDA-approved chemotherapeutic agents. A research performed on the FDA clinical trials website found eight phase 1 ABT-888 combination trials, six of which currently recruiting, spanning from glioblastoma therapy to non-Hodgkin lymphoma to CML, and to solid tumours such as breast and ovarian cancer, and melanoma [14]. Gordon and Collins therefore agreed that the primary goal of a phase 0 trial must be the evaluation of target modulation, which has to inform decision making for further drug developments and for pipeline prioritization decisions. Two different requirements are both fundamentally important in this respect, as Gordon underscored: (1) the choice of a good drug candidate for a phase 0 trial, as not all agents are suitable candidate drugs [15], (2) the willingness to stop the drug under scrutiny if there are no definitive answer to the questions raised. Only if both requirements are satisfied will phase 0 cancer trials fulfil their expectations to speed up the drug development process and to cut costs.

A European perspective on the nascent field was given by JHM Schellens (The Netherlands Cancer Institute, Amsterdam), who explained how the EMEA guidelines provide flexible guidelines for phase 0 trials and touched upon the issue of feasibility [2]. Interestingly, while FDA’s guidance does not issue new regulation, but is an interpretation of existing recommendations on drug development—stressing how limited, early first-in-human studies are often supported by a more extensive pre-clinical database than required by regulation—the EMA Position paper talks about a new kind of non-clinical safety studies needed to support human clinical trials with a single dose of a pharmacologically active compound [16]. Shellens provided examples of drugs for which a phase 0 approach would have worked, had the guidelines been there a few years ago. A good example of a phase 0 candidate was EO9, a bioreductive agent with an excellent pre-clinical *in vivo* activity [17], or SPI-77, a formulation of cisplatin within a liposome [18]. According to Schellens, phase 0 studies are not entirely new (for a similar opinion read [19]), and in support of his claim, he quoted a 1994 study with capecitabine where, prior to the phase 1 trial, a PK comparison was made between two almost identical chemical structures in a clinical ADME study [20]. Schellens concluded his speech with some critical remarks and challenges to feasibility that must be kept in mind when planning a phase 0 trial, among others: the presence or not of a linear pharmacokinetic for the drug, the availability of a sensitive bioanalytical method and of a sufficiently qualified research team and adequate infrastructure, the careful examination of the drug candidates, the availability of a measurable PD effect at very low doses, the feasibility of tumour tissue sampling with related ethical considerations, the choice of good patient candidates. ‘Phase 0 will not solve all our problems’, Schellens argued, ‘but they can solve some of our problems, as they can help identify optimal clinical candidate among a series of similar agents and may save time and money’. But, he emphasized, ‘phase 0 trials will not solve any problem if there is a lack of pre-clinical proof of principle of the drug mechanism of action, or if a good biomarker is absent, or if the PK is very complex or the linearity of the PD is not established’ [16].

At the end of the day, the phrase ‘phase 0 trials’ looks like a big umbrella covering maybe too many things: Microdose studies, functional imaging studies, molecular proof-of-concept studies, which briefly reach a pharmaceutical dose. Is there room for improvement in the current drug pipeline, for example in the incorporation of phase 0 trials in a phase 1 studies, as Schellens proposed, or only for a drastic re-engineering of the drug development process, as Collins argued?

Wouldn’t it be more appropriate to call phase 0 trials only those studies where no pharmaceutical therapeutic dose is ever reached, as microdose studies and imaging trials, for which pre-clinical animal studies requisites would remain very low, and the risk of toxicity too? And how many research centres in the world would actually meet the high standards required to perform a phase 0 trial?

It is still too early to provide such answers. Only in a few years we will be able to respond to these issues, and to the initial point posed by James Doroshow, whether phase 0 trials are really necessary. Most importantly, we should not give for granted the participation of the patients in trials where no direct benefit is expected, and who would be motivated only by an out-of-the-ordinary altruism.
